# Additional Effects of Facilitatory Cerebellar Repetitive Transcranial Magnetic Stimulation on Inhibitory Repetitive Transcranial Magnetic Stimulation over the Unaffected Contralesional Primary Motor Cortex for Motor Recovery in Subacute Ischemic Stroke Patients

**DOI:** 10.3390/jcm14072315

**Published:** 2025-03-28

**Authors:** Sungwon Kim, Ho Seok Lee, Heegoo Kim, Dae Hyun Kim, Won Hyuk Chang

**Affiliations:** 1 Department of Physical and Rehabilitation Medicine, Center for Prevention and Rehabilitation, Heart Vascular Stroke Institute, Samsung Medical Center, Sungkyunkwan University School of Medicine, Seoul 06351, Republic of Korea; sungwon11.kim@samsung.com (S.K.); hoseok89.lee@samsung.com (H.S.L.); hiheegoo@gmail.com (H.K.); hohoho7490@gmail.com (D.H.K.); 2 Department of Health Sciences and Technology, Samsung Advanced Institute for Health Science and Technology, Sungkyunkwan University, Seoul 06355, Republic of Korea

**Keywords:** repetitive transcranial magnetic stimulation, stroke, motor learning, cerebellum, rehabilitation

## Abstract

**Background/Objectives**: Cerebellum might be one of the targets of repetitive transcranial magnetic stimulation (rTMS) for motor recovery in stroke patients. The aim of this study was to investigate the enhancing effects of rTMS over the cerebellum on inhibitory rTMS for motor recovery in patients with subacute ischemic stroke. **Methods**: Twenty-three patients with subacute ischemic stroke were recruited into this single-blind randomized, controlled study with a blinded observer. The Cr-Cbll group received Cr-Cbll rTMS consisting of continuous theta burst stimulation over the contralesional primary motor cortex (M1), a shoulder mobilization exercise, and high-frequency rTMS over the contralesional cerebellum. The Cr-sham group received sham rTMS over the cerebellum instead of high-frequency rTMS. All participants received ten daily sessions for 2 weeks. The Fugl-Meyer Assessment (FMA) was measured before, immediately after, and 2 months after the intervention. **Results**: A total of 20 participants (10 in the Cr-Cbll group and 10 in the Cr-sham group) who completed the two-week intervention were included in the intention-to-treat analysis. There was no significant difference in general and clinical characteristics between the two groups at baseline. Total and upper extremity scores of FMA showed a significant interaction between time and group (*p* < 0.05). Each improvement of upper extremity score of FMA immediately and 2 months after the intervention was significantly higher in the Cr-Cbll group than in the Cr-sham group (*p* < 0.05). **Conclusions**: These results demonstrated that rTMS over the cerebellum could have additional effects on inhibitory rTMS over contralesional M1 for improving upper extremity motor function in patients with subacute ischemic stroke.

## 1. Introduction

Repetitive transcranial magnetic stimulation (rTMS) has been demonstrated to exert either inhibitory or facilitatory effects on cortical excitability. These modulatory effects are dependent on the stimulation frequency [1]. With the loss of function of the ipsilesional hemisphere in stroke patients, there are changes in the interaction between the ipsilesional hemisphere and the contralesional hemisphere via the corpus callosum [2]. Based on this theory, many studies have reported that low-frequency rTMS or continuous theta burst stimulation (cTBS) applied to the contralesional primary motor cortex (M1) can improve upper extremity function [3,4]. Currently, the most common rTMS strategy to improve motor function in patients with ischemic stroke is to inhibit unaffected M1 [3]. Although this rTMS strategy is known to be a promising therapeutic tool, the effect size for improving motor function in stroke patients has been relatively small [3].

A number of previous studies have reported that the cerebellum is involved in motor learning in conjunction with other critical areas, including M1 [5,6,7,8]. Consequently, these studies have indicated that the cerebellum may be a viable target for rTMS in the motor recovery of stroke patients, but the evidence is limited. Two sham-controlled studies including a randomized controlled trial investigating the effects of cerebellar rTMS on motor performance showed only modest improvements [9,10]. Since inhibitory rTMS over unaffected M1 has been shown to improve motor recovery in stroke patients, combining it with an additional cerebellar rTMS may have enhancing effects on motor recovery.

We hypothesized that an additional contralesional cerebellar rTMS would augment the effects of inhibitory rTMS over unaffected M1 in patients with subacute ischemic stroke. The effects of facilitatory rTMS on patients with ischemic stroke are unclear when there is no respond to transcranial magnetic stimulation-induced motor-evoked potentials (TMS-induced MEPs). Therefore, inhibitory rTMS of the unaffected M1 is a proven effective method in these ischemic stroke patients [11]. Furthermore, the TMS-induced MEP response of the affected M1 is a crucial factor in predicting the prognosis of motor function improvements and the efficacy of rTMS in patients with ischemic stroke [12]. Consequently, patients without the MEP response were selected as participants for this study. The objective of this study was to examine the potential benefits of cerebellar rTMS on conventional inhibitory rTMS for motor recovery in subacute ischemic stroke patients with no response to TMS-induced MEPs.

## 2. Materials and Methods

### 2.1. Participants

Participants with subacute hemiparetic ischemic stroke were enrolled at an inpatient clinic in the rehabilitation department of a tertiary hospital in South Korea. Inclusion criteria were as follows: (1) hemiparetic cerebral infarction, (2) post-onset duration of less than one month, (3) moderate to severe motor impairment (Fugl-Meyer Assessment (FMA) score < 85) [13], (4) no response to TMS-induced MEPs of ipsilesional M1, (5) cognitive and language functions to perform more than one level of command, and (6) age of 19 years or older. Exclusion criteria were as follows: (1) contraindications to rTMS such as a history of seizures, the presence of intracranial metallic implants, or the surgical placement of medical devices, and so on [14]; (2) progressive or unstable stroke; (3) pre-existing and active major neurological or major psychiatric disease; and (4) pregnant or lactating women.

The methods employed in this study were reviewed and approved by the Samsung Medical Center Institutional Review Board (2020-06-068). The study was conducted in accordance with the 2013 WMA Declaration of Helsinki. Furthermore, the study was conducted with the understanding and written consent of each participant.

### 2.2. Study Design

This study was a single-blind, randomized, parallel, sham-controlled trial with a blinded observer. Participants were randomized into two groups: the cerebrum-cerebellum (Cr-Cbll) group or the cerebrum-sham (Cr-sham) group. The randomization process was carried out by an investigator who was not involved with the participants and utilized an online random allocation tool. The participant allocation ratio for each group was 1:1. The blinding method of this study was single-blind with the blind observer clinical trial, which means that the person performing the intervention knew the participant’s group, but the person performing the assessments or the participant himself did not know the participant’s group. For the intervention, the Cr-Cbll group received Cr-Cbll rTMS, and the Cr-sham group received Cr-sham rTMS. Motor function assessments were performed before (T0), immediately after (T1), and 2 months after the intervention (T2). During the intervention, participants in both groups received the same amount of conventional inpatient stroke rehabilitation.

### 2.3. Determination of the MEP Response and Stimulation Location

TMS-induced MEPs were assessed separately via single magnetic stimulations at 120% of the resting motor threshold (rMT) over each M1 using a 70 mm figure-of-eight coil. The participant was comfortably seated in an armchair with their eyes open during the experiments. A Synergy electromyography/evoked potential system (Medelec Co., Ltd., Kingswood, Bristol, UK) was used to record and monitor the activity of the contralateral first dorsal interosseus (FDI) muscle. Single-pulse TMS was applied over the affected M1 using a Magstim Rapid2^®^ stimulator (Magstim Co., Ltd., Spring Gardens, Whitland, Carmarthenshire, Wales, UK) equipped with a 70 mm figure-of-eight coil. The coil was held tangentially to the scalp with the handle pointing posteriorly and laterally at 45° from the mid-sagittal line. For TMS, the optimal position (“hot spot”) was defined as the location where TMS elicited MEPs of maximum peak-to-peak amplitude in the contralateral FDI muscle. rMT was defined as the lowest stimulus intensity capable of eliciting MEPs of at least a 50 µV peak-to-peak amplitude on 5 out of 10 consecutive trials. Five sweeps of MEPs at 120% of rMT were collected [15]. If no EMG activity was detected in 10 trials at 90% stimulator power, the participant was considered to have no MEP response.

### 2.4. rTMS Intervention

The experimental protocol comprised ten daily intervention sessions, which were administered over a period of two weeks to all participants. rTMS was delivered on the scalp over the unaffected M1 in accordance with safety recommendations [16] using a figure-of-eight coil connected to a Magstim Rapid^®^ stimulator with two booster modules (Magstim Co., Ltd., Spring Gardens, Whitland, Carmarthenshire, Wales, UK). The Cr-Cbll rTMS consisted of three steps, starting with cTBS over the unaffected M1 for 40 s at 70% of the rMT of the unaffected M1. This was followed by a motor task involving the mobilization of the affected shoulder for 10 min. Finally, high-frequency rTMS was applied over the contralesional cerebellum for 10 min. The participants in this study demonstrated no response to TMS-induced MEPs, which consequently rendered the majority of participants unable to perform active exercises on their hands and forearms. Therefore, shoulder mobilization was performed to provide the same exercise therapy. Cr-sham rTMS was identical to the first two steps but included sham rTMS using a sham coil [17] over the contralesional cerebellum instead of high-frequency rTMS. cTBS consisted of a burst of 3 pulses at 50 Hz, with an intensity of 70% RMT of the unaffected M1, repeated at 200 ms intervals, for a total of 600 pulses delivered over 40 s. High-frequency rTMS over the contralesional cerebellum consisted of 20 cycles, and each cycle consisted of 50 pulses at 10 Hz for 5 s and 25 s of rest at an intensity of 100% rMT of the unaffected M1, for a total of 1000 pulses delivered. The stimulation site was 3 cm lateral to the palpated external occipital protuberance, and the coil was positioned tangential to the scalp with the handle pointing superiorly [18,19,20].

### 2.5. Baseline Characteristics and Outcome Measures

The medical records of the participants were collected, and the following data were documented: age, sex, type of stroke, side of stroke, and duration after onset. For all participants, motor function domain scores of the Fugl-Meyer Assessment were determined [21]. The total, upper extremity, and lower extremity scores of the FMA (total FMA, FMA-UE, and FMA-LE) were assessed separately. The Functional Ambulatory Category (FAC) [22] was also used to assess for mobility and gait function. All assessments were carried out by a single licensed occupational therapist who was unaware of the participant’s group allocation. Throughout the intervention study period, we planned to record all symptoms potentially related to the intervention, including seizures, headaches, neck discomfort, paresthesia, changes in hearing or vision, and syncope.

### 2.6. Comparative Analysis on Brain Lesions

To compare the location and extent of the brain lesions of the participants in each group, we collected their axial diffusion-weighted imaging sequence MRI scans and analyzed the lesion with lesion overlap mapping. The initial axial diffusion-weighted imaging (DWI) scans of each participant were acquired from our institution’s picture archiving and communication system in the digital imaging and communications in medicine (DICOM) data format. The DICOM data were then converted to the Neuroimaging Informatics Technology Initiative (NIFTI) format. Dcm2niix, an open-source software (https://github.com/rordenlab/dcm2niix, version 1.0.20230411, accessed on 28 January 2024) designed to convert neuroimaging data from DICOM to the NIFTI format, was used for this conversion. Next, lesion segmentation from the original DWI scans was performed using the 3D Slicer. The 3D Slicer (https://www.slicer.org, version 5.6.0, accessed on 28 January 2024) is a free, open-source software for the visualization, processing, segmentation, and analysis of three-dimensional medical images. Finally, we created an overlap lesion map for each group, which contains overlay images of the affected side’s stroke lesions of the participants in each group. This process was performed using MRIcron. The MRIcron software (https://www.nitrc.org/projects/mricron, version 1.0.20190902, accessed on 28 January 2024) is a cross-platform NIFTI-format image viewer that can load multiple image layers and draw overlap images. The resulting overlap lesion maps were qualitatively compared by the investigators.

### 2.7. Statistical Analysis

The primary outcome of this study was the difference in the upper extremity FMA score (FMA-UE, range: 0–66) from T0 to T1. To calculate the required sample size for the study, we employed Lehr’s formula [23]. The desired significance level (α) was set at 0.05, and the desired power (1-β) was set at 0.90. Clinically significant differences in the primary outcome were determined to be 12.40, based on a preceding study [24]. The standard deviation was estimated to be 7.70, and the follow-up rate was set at 80.0%; these values were derived from a prior study involving participants with analogous characteristics [12]. This process resulted in the determination that the recruitment of more than 22 participants was necessary for the study, with 11 participants allocated to each group.

The full analysis set was defined as the population of participants who underwent functional assessments at least at baseline (T0) and immediately after the intervention (T1). Missing data were imputed using the last observation carried forward (LOCF) method. To compare the baseline demographic and clinical characteristics of the two groups, an independent t-test was used for continuous variables, based on the assumption of a normal distribution, which was confirmed by the Shapiro–Wilk test (*p* > 0.05). Chi-squared test was used for categorical variables. Repeated measures analysis of variance (ANOVA) was used to perform the analysis between groups and to verify the presence of an interaction between group and time. Post hoc analyses were performed using paired *t*-test for within-group analysis and independent t-test for between-group analysis. Bonferroni’s correction was employed to address the issue of multiple comparisons. Statistical significance was defined as a *p*-value less than 0.05.

## 3. Results

### 3.1. Participants Characteristics

**Figure 1:**
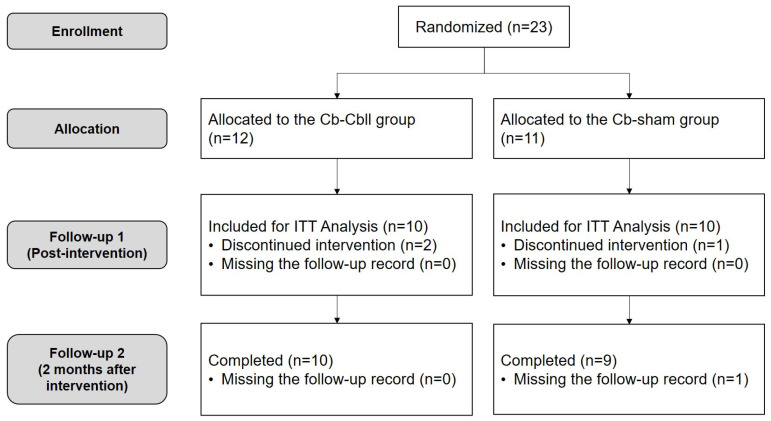
Flowchart of the study; ITT: intention-to-treat.

In this study, 23 participants were enrolled and subsequently divided into two groups: 12 in the Cr-Cbll group and 11 in the Cr-sham group. Three patients withdrew during the intervention for personal reasons unrelated to the intervention, and one patient missed the last follow-up visit. The intervention was well tolerated, and no adverse effects were reported by 20 participants. Finally, 20 participants (10 in the Cr-Cbll group and 10 in the Cr-sham group) completed the intervention for 2 weeks and were included in the intention-to-treat analysis (Figure 1).

**Figure 2:**
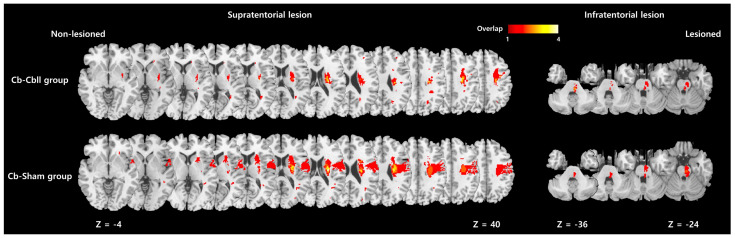
Overlap lesion maps in each group; there was no significant difference in stroke lesion between the two groups. z: z-axis in the Montreal Neurological Institute space.

A comparison of the two groups at T0 revealed no statistically significant disparities in demographic and clinical characteristics, including motor function (Table 1). Overlap lesion maps were generated for each group (Figure 2). Both groups included lesions in the primary motor cortex, corona radiate, midbrain, midbrain, and pons associated with the corticospinal tract. The corona radiate was the most overlapped lesion in both groups. The cerebellum was not involved in the overlap lesion maps of either group.

### 3.2. Changes in the Fugl-Meyer Assessment

**Figure 3:**
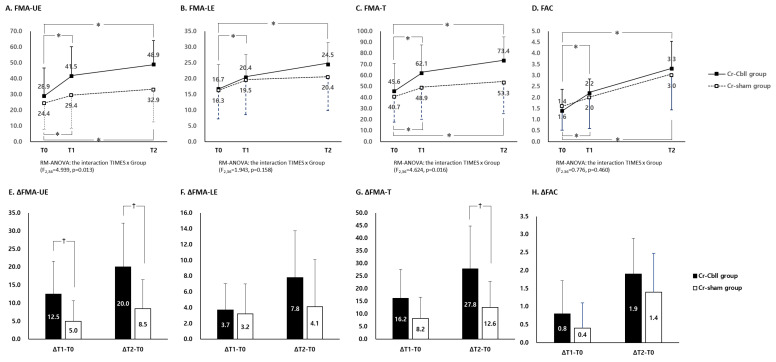
Changes in Fugl-Meyer Assessment scores and the Functional Ambulatory Category. Measurements were performed prior to treatment (T0), immediately after repetitive transcranial magnetic stimulation (rTMS) (T1), and 2 months after rTMS (T2). The error bars represent the standard deviation for each group. FMA, Fugl-Meyer Assessment; FMA-UE, upper extremity score of the FMA, FMA-LE, lower extremity score of the FMA; FMA-T, total score of the FMA, FAC; Functional Ambulatory Category. * *p* < 0.05, compared with the value at T0. ^†^ *p* < 0.05, a comparison between the two groups.

FMA-UE scores showed a significant interaction between time and group (time × group interaction: F_2,36_ = 4.939 and *p* = 0.013). FMA-UE at T1 and T2 in each group showed a significant improvement compared to that at T0 (*p* < 0.05, Figure 3A). The improvements of FMA-UE from T0 to T1 were 12.5 ± 9.1 and 5.0 ± 5.4 in the Cr-Cbll group and the Cr-sham group, respectively. Each improvement of FMA-UE from T0 to T1 and from T0 to T2 was significantly higher in the Cr-Cbll group than in the Cr-sham group (*p* < 0.05, Figure 3E). Furthermore, the number of participants who reached the minimal clinically important difference (MCID) for FMA-UE [25] at T1 was seven at T1 and eight at T2 in the Cr-Cbll group. In the Cr-sham group, the number was four at T1 and five at T2. These results show a higher tendency in the Cr-Cbll group, although this was not statistically significant (*p* = 0.370 and *p* = 0.350).

FMA-LE scores showed no significant interaction between time and group (time × group interaction: F_2,36_ = 1.943 and *p* = 0.158). FMA-LE at T1 and T2 showed a significant improvement only in the Cr-Cbll group compared to that at T0 (*p* < 0.05, Figure 3B). The improvements of FMA-LE from T0 to T1 and from T0 to T2 showed no significant difference between the two groups (Figure 3F). The number of participants who reached the MCID for FMA-LE [26] at T1 was two in the Cr-Cbll group and six in the Cr-sham group. At T2, the number was four in both groups. There was no significant difference at T1 and T2 between the two groups.

FMA-T scores showed a significant interaction between time and group (time × group interaction: F_2,36_ = 4.624 and *p* = 0.016). FMA-T at T1 and T2 in each group showed a significant improvement compared with that at T0, respectively (*p* < 0.05, Figure 3C). The improvement of FMA-T from T0 to T1 showed no significant difference between the two groups. However, the improvement of FMA-T from T0 to T2 was significantly higher in the Cr-Cbll group than in the Cr-sham group (*p* < 0.05, Figure 3G). The number of participants who reached the MCID for FMA-T [27] at T1 was four in the Cr-Cbll group and two in the Cr-sham group. At T2, the number was nine in the Cr-Cbll group and five in the Cr-sham group. The Cr-Cbll group demonstrated a higher tendency at T2 without statistical significance (*p* = 0.141).

Changes in FMA subscales are shown in Table 2. FMA-UE-A scores showed a significant interaction between time and group (time × group interaction: F_2,36_ = 6.311 and *p* = 0.004). FMA-UE-A at T1 showed a significant improvement compared to T0, but only in the Cr-Cbll group (*p* < 0.05). FMA-UE-A at T2 in each group showed a significant improvement compared to that at T0. Each improvement of FMA-UE-A from T0 to T1 and from T0 to T2 was significantly higher in the Cr-Cbll group than in the Cr-sham group (*p* < 0.05). However, the scores of the other FMA subscales showed no significant interaction between time and group.

### 3.3. Changes in Functional Ambulatory Category

FAC showed no significant interaction between time and group (time × group interaction: F_2,36_ = 0.776 and *p* = 0.460). FAC at T1 and T2 in each group showed a significant improvement compared to that at T0 (*p* < 0.05, Figure 3D). There was no significant difference in the improvement of FAC from T0 to T1 and from T0 to T2 between the two groups (Figure 3H).

## 4. Discussion

The results of this study demonstrated that cerebellar rTMS over the contralesional cerebellum has additional immediate and long-term effects on inhibitory rTMS over the contralesional M1 for improving the upper extremity motor function of the affected side in subacute ischemic stroke patients with no TMS-induced MEP response. These improvements in upper extremity motor function were mainly observed in the subscale of the FMA-UE-A, which represents proximal upper extremity function, including the shoulder, elbow, and forearm.

Motor learning is crucial for motor recovery in stroke patients [28,29]. Studies using serial functional magnetic resonance imaging (fMRI) during motor learning or motor adaptation tasks have demonstrated an initial activation and subsequent decrease in activity in the cerebellar cortex, followed by an increase in activity in the anteromedial cerebellar nuclei. These findings suggest a potential involvement of the cerebellum in both early motor learning and consolidation processes [7,30,31]. A longitudinal fMRI study reported the involvement of bilateral striatum and the contralesional cerebellum during the acute phase of stroke recovery [32]. These findings suggest that the cerebellum may be a novel target for rTMS because of its important role in the motor learning process. Recently, attempts have been made to modulate cerebellar activity with rTMS [33]. One study reported that low-frequency rTMS applied to the medial cerebellum of healthy participants caused increased variability in a paced finger tapping task [34]. Another study on healthy participants revealed that low-frequency repetitive transcranial magnetic stimulation (rTMS) administered over the right paramedian cerebellum led to impaired performance on the 10-hole pegboard task [35]. Torriero et al. [36] demonstrated that low-frequency rTMS applied to the lateral cerebellum impaired performance on the serial reaction time task, which represents procedural learning. These studies suggest that low-frequency rTMS may negatively alter cerebellar function, which is important for upper extremity motor performance and motor learning. These studies are consistent with our findings, highlighting that cerebellar rTMS has an enhancing effect on conventional inhibitory rTMS for motor recovery in subacute stroke patients.

The role of the cerebellum in motor function is another hypothesis that could explain the results of this study. The cerebellum is particularly involved in controlling proximal upper extremity movements [37,38]. The cerebellum has important connections to nuclei in the brainstem that are the origin of descending pathways that control the proximal musculature [39]. These roles of motor function in the cerebellum may explain why the effects of cerebellar rTMS were clearly seen in FMA-UE, especially in FMA-UE-A, in this study. In addition, this study was conducted in stroke patients with a negative response to TMS-induced MEPs. The corticospinal tract is responsible for controlling movement, particularly in the upper extremities, and the structural integrity of the corticospinal tract is critical for motor recovery in stroke patients [40,41,42]. In patients with impaired corticospinal tract integrity after stroke, the cerebellum and its connections may be involved in promoting motor learning. Several studies have shown that the activity of the cortico-cerebellar system can be modulated by various modalities of non-invasive cerebellar stimulation [33]. Cortico-cerebellar connections, including the dentatothalamocortical tract, have been demonstrated to be associated with the prognosis of motor function in stroke patients [43,44,45]. Yoo et al. [45] analyzed diffusion tensor imaging with subacute ischemic stroke and concluded that the microstructural integrity of the dentatothalamocortical tract is a predictor of chronic upper extremity motor function, independent of the integrity of the corticospinal tract. In light of these prior findings and the results of the present study, it can be concluded that subacute ischemic stroke patients with severe damage to the corticospinal tract may be suitable candidates for cerebellar rTMS to enhance proximal upper extremity motor function.

The effect of rTMS over cerebellum in this study might be attributable to the involvement of the cerebellum in the mirror neuron system. Recent studies suggest that the cerebellum is also involved in cognitive and social functions, including those related to the mirror neuron system [46,47]. The cerebellum may play a role in a mirror-matching mechanism by simulating actions internally, even in circumstances where such actions are merely observed [46]. The process under investigation involves the cerebellum’s connections with other brain regions, thereby facilitating visuomotor matching and learning through imitation [47]. Neuromodulation by rTMS in stroke patients is well documented [3]; however, there is few studies on the possibility of true recovery after stroke. However, it is hypothesized that true recovery might be achieved through the effect of the cerebellum rTMS lesion network, which is not directly related to the lesion. The employment of advanced neuroimaging techniques, such as functional MRI and diffusion tensor imaging, is expected to provide further important insights into the underlying mechanisms. TMS-induced MEPs are a neurophysiological assessment tool that can evaluate the excitability of the corticospinal tract. In this study, a subsequent TMS-induced MEP evaluation was not conducted following the baseline evaluation. This constitutes a significant limitation of the present study, and it is anticipated that further study will be needed to investigate the neurophysiological mechanisms of rTMS over the cerebellum.

The cerebellum coordinates gait and maintains posture. The previous study of six patients with cerebellar stroke showed that excitatory patterned rTMS over the damaged lateral cerebellum resulted in improvements in postural and gait assessments [48]. Koch et al. [10] showed that excitatory patterned rTMS over the contralesional cerebellum promoted improvements in gait and balance in the intervention group compared to the control group. In this study, there was no significant effects of additional cerebellar rTMS on ambulatory function, as assessed by FAC. FAC is a relatively crude assessment tool for ambulatory function, and a relatively small number of participants in this study may explain this discrepancy. A more precise assessment, such as the Timed Up and Go Test or the Berg Balance Scale, can provide a more accurate evaluation of a patient’s balance and gait function. Future studies with more precise balance and gait assessment tools will be needed.

In comparison with preceding studies [12,49], a greater number of enhancements in motor function were observed in both the Cb-Cbll and Cb-sham groups. This can be attributed to the recruitment criteria, which necessitated the inclusion of participants exhibiting some degree of preserved cognitive and language function. Consequently, the study population included a higher proportion of individuals with smaller infarct lesion size. Consequently, the findings of this study cannot be extrapolated to determine the effects of rTMS on all ischemic stroke patients. However, no significant change was observed in the subscale of FMA-UE in the Cb-sham group. These findings could be due to the limited number of participants. It should be noted that the results of this study alone are insufficient to provide a definitive clarification of the previously reported outcomes concerning the effects of inhibitory rTMS over unaffected M1 [3]. In this study, no significant differences in overlap lesion maps were observed between the two groups, including midbrain and pons lesions. This suggests that the results are unlikely to be influenced by confounding factors. However, it is important to note that midbrain and pons lesions, particularly those affecting the cerebellar peduncle, have the potential to influence the outcomes of rTMS over the cerebellum. Further study with a large number of participants will be needed to compare the differences in effects between infratentorial and supratentorial lesions.

Interhemispheric inhibition has been demonstrated to exert a substantial influence on the process of motor recovery after stroke by influencing the balance between the hemispheres. Appropriate interventions that target this imbalance can enhance motor recovery outcomes [3]. However, the effectiveness of interventions targeting interhemispheric inhibition can vary significantly among stroke patients, highlighting the need for personalized treatment approaches [50]. In this study, all participants received inhibitory rTMS to the unaffected M1 with rTMS to the cerebral cortex; however, this method may not be the most appropriate rTMS method for all stroke patients. The development of reliable methods to measure interhemispheric inhibition and its changes over time is crucial for the optimization of rehabilitation strategies [51]. In the application of rTMS to subacute ischemic stroke patients, it has been suggested that it would be better to assess individual differences such as interhemispheric imbalance and apply rTMS accordingly.

This study has several limitations. First, the stimulation site of cerebellar rTMS was determined using the lateral distance from the palpated external protuberance. Anatomical variability between subjects or subjective palpation by the operator may have influenced the results of cerebellar rTMS. The validated sham coil was utilized in the Cr-sham group to induce sham stimulation. However, since the validation of the sham coil was for cerebral stimulation, the lack of research on the usefulness of the sham coil for cerebellar stimulation was considered to be a limitation of this study. In addition, participants were not controlled for quantitative and qualitative aspects of conventional rehabilitation after the interventional period. Physical or occupational therapy measures applied after the intervention might have influenced the results. The LOCF method employed in this study has the potential to underestimate the treatment effect, particularly in cases where the function exhibits natural improvements over time, as observed in this study’s participants. The dropout of a patient at two months after the intervention in the control group may have led to an underestimation of the long-term intervention effect in the control group. This is one of the limitations in this study.

## 5. Conclusions

This study demonstrated that cerebellar rTMS over the contralesional cerebellum has additional effects compared to inhibitory rTMS over contralesional M1 for improving upper extremity motor function in subacute ischemic stroke patients without a TMS-induced MEP response, mainly improving proximal upper extremity function. This study might inform novel rTMS strategies for improving upper extremity motor function in subacute stroke patients, who have a poor prognosis for recovery of upper extremity motor function.

## Figures and Tables

**Table 1:** Baseline demographic and clinical characteristics of the participants (ITT analysis).

	Cr-Cbll Group (*n* = 10)	Cr-Sham Group (*n* = 10)	*p*-Value
Age (years)	68.2 ± 5.2	67.2 ± 11.7	0.809
Sex (male/female)	8:2	6:4	0.628
Stroke type (first-ever/recurrent)	10:0	8:2	0.474
Stroke side (right/left)	6:4	4:6	0.638
Stroke lesion (supratentorial/infratentorial)	7:3	7:3	1.000
Stroke duration (days)	12.1 ± 4.7	15.1 ± 6.5	0.251
rMT of affected M1	-	-	-
rMT of unaffected M1	55.4 ± 12.0	46.6 ± 12.9	0.132
Fugl-Meyer Assessment			
FMA-UE	28.9 ± 17.7	24.4 ± 16.5	0.564
FMA-LE	16.7 ± 7.7	16.3 ± 7.1	0.906
FMA-T	45.6 ± 25.2	40.7 ± 22.9	0.655

Values are presented as mean ± SD. rMT, resting motor threshold; M1, primary motor cortex; FMA, Fugl-Meyer Assessment; FMA-UE, upper extremity score of the FMA; FMA-LE, lower extremity score of the FMA; FMA-T, total score of the FMA.

**Table 2:** Changes in subscales of the Fugl-Meyer Assessment (ITT analysis).

		T0	T1	T2	ΔT1-T0	ΔT2-T0
FMA-UE-A	Cr-Cbll group (n = 10)	17.2 ± 9.2	23.9 ± 9.4 *	27.7 ± 7.1 *	6.5 ± 5.2 ^†^	10.4 ± 5.7 ^†^
	Cr-sham group (n = 10)	15.7 ± 9.1	17.9 ± 11.0	19.6 ± 10.6 *	2.2 ± 2.7	3.9 ± 3.7
FMA-UE-B	Cr-Cbll group (n = 10)	3.8 ± 3.1	5.5 ± 3.1 *	7.2 ± 2.7 *	1.7 ± 2.1	3.4 ± 2.8
	Cr-sham group (n = 10)	2.4 ± 2.6	3.0 ± 2.6	3.8 ± 2.8 *	0.6 ± 1.7	1.4 ± 2.5
FMA-UE-C	Cr-Cbll group (n = 10)	5.7 ± 4.2	8.7 ± 4.6 *	10.7 ± 4.3 *	3.0 ± 2.4	5.0 ± 3.9
	Cr-sham group (n = 10)	4.4 ± 3.4	6.2 ± 5.8	7.6 ± 5.7 *	1.8 ± 2.9	3.2 ± 2.7
FMA-UE-D	Cr-Cbll group (n = 10)	2.1 ± 1.9	3.4 ± 1.9 *	3.3 ± 2.2 *	1.3 ± 1.3	1.2 ± 1.5 ^†^
	Cr-sham group (n = 10)	1.9 ± 2.0	2.3 ± 2.1	1.9 ± 2.0 *	0.4 ± 1.0	0.0 ± 0.5
FMA-LE-E	Cr-Cbll group (n = 10)	14.4 ± 5.9	17.2 ± 5.3 *	20.6 ± 5.7 *	2.8 ± 1.9	6.2 ± 4.6
	Cr-sham group (n = 10)	13.3 ± 5.2	15.9 ± 6.9	16.6 ± 7.7	2.6 ± 3.1	3.3 ± 4.8
FMA-LE-F	Cr-Cbll group (n = 10)	2.3 ± 2.1	3.2 ± 2.3	3.9 ± 1.4	0.8 ± 2.0	1.6 ± 2.0
	Cr-sham group (n = 10)	3.0 ± 2.1	3.6 ± 2.0	3.8 ± 2.0	0.6 ± 1.6	0.8 ± 1.5

Values are presented as mean ± SD. FMA-UE-A, upper extremity subscale (A) of the FMA-UE; FMA-UE-B, wrist subscale (B) of the FMA-UE; FMA-UE-C, hand subscale (C) of the FMA-UE; FMA-UE-D, coordination/speed subscale (D) of the FMA-UE; FMA-LE-E, lower extremity subscale (E) of the FMA-LE; FMA-LE-F, coordination/speed subscale (F) of the FMA-LE. * *p* < 0.05, compared with the value at T0. ^†^ *p* < 0.05, compared with the value of the Cr-sham group.

## Data Availability

The data presented in this study are available upon reasonable request from the corresponding author. The data are not publicly available due to privacy and ethical restrictions.
